# Reducing carbon emissions in the healthcare sector: a real-time delphi on challenges and enablers

**DOI:** 10.1080/11287462.2026.2668168

**Published:** 2026-05-06

**Authors:** Valentina de Maack, Sandy Tubeuf, Nathalie Clavel, Isabelle Ethier, Stephan Williams, Pierre-Marie David, Charles Dupras

**Affiliations:** a École de santé publique de l’Université de Montréal, Montréal , Québec, Canada; b Institut de Recherche Santé et Société – Université catholique de Louvain – Faculté de Santé Publique , Brussels, Belgique; c Division of Nephrology, Department of Medicine, Centre hospitalier de l’Université de Montréal, Montréal, Québec, Canada; d Health innovation and evaluation hub, Centre de recherche du Centre hospitalier de l’Université de Montréal, Montréal, Québec, Canada; e Département d’anesthésiologie, Centre hospitalier de l’Université de Montréal (CHUM) , Montréal, Québec, Canada; f Faculté de pharmacie de l’université de Montréal – 2940 Chem. de Polytechnique, Montréal, Québec, Canada

**Keywords:** Green health care, Ethics, Trade-offs, Sustainability, Carbon emissions, Challenges, Enablers

## Abstract

Healthcare systems are increasingly committing to climate action, yet efforts to reduce their environmental footprint raise operational challenges. Decarbonization initiatives can generate concerns about cost, patient safety, care quality, and equitable access, revealing tensions that complicate implementation. This study examines how experts assess the impact and feasibility of climate-related interventions in healthcare, identifying key challenges and enablers. Using a Real-time Delphi approach, we consulted 14 specialists with ethics, sustainability, biomedical sciences, and public health backgrounds, who evaluated the relevance and applicability of five *social tipping elements* within the healthcare context. Interventions targeting energy systems and infrastructure were viewed as potentially high-impact but limited by regulatory complexity, infrastructural constraints, and ethical concerns. In contrast, strategies centered on education and shifts in values were perceived as more feasible, though questions about operationalization remain. Experts also noted potential issues, such as patient mistrust or clinician disengagement, if environmental goals are seen as misaligned with core commitments of healthcare practice. Overall, these findings underscore the need for inclusive, ethically robust, evidence-informed, and context-sensitive strategies that integrate sustainability goals with patient, professional, and institutional priorities. Improving access to environmental impact data and supporting shared decision-making emerged as key enablers for fostering acceptable ecological transitions in healthcare.

## Introduction

Healthcare systems contribute significantly to environmental degradation (Lenzen et al., 2020), making their ecological transition increasingly urgent (Alami et al., 2023). While the environmental and economic impacts of healthcare systems are becoming better documented (Keil et al., 2024; Scapecchi, 2008), the ethical and social dimensions raised by this transition remain insufficiently examined. Several factors heighten the likelihood of tensions emerging. Quantitative data on the environmental impact (EI) of many treatments and technologies are limited. Ethical guidance is also lacking (de Maack et al., 2024; Toccalino et al., 2021), placing professionals at the forefront of decisions that involve weighing environmental costs alongside clinical considerations. Moreover, little is known about how healthcare-related carbon emissions are perceived relative to other sources of emissions, or about stakeholders’ moral readiness to integrate environmental considerations into their practices and decision-making.

While evidence suggests that well-designed sustainable interventions can often support both patient care and sustainability goals (Alim & Sulley, 2024), there are cases where these aims do not or do not seem to align naturally (de Maack et al., 2024; Parker, 2023). We characterise these as *trade-off situations*, where environmental considerations enter deliberation as one good among others and are weighed against clinical goods and institutional constraints (Holtorf et al., 2025). The resulting balance reflects context-specific values and ethical judgement rather than a single correct solution (Pegg et al., 2024). These situations, which showcase seemingly competing goals or the need to balance them, have already been discussed in the health technology assessment (HTA) literature on the integration of environmental criteria (Holtorf et al., 2025). They are also addressed in specific clinical domains, for example, general anaesthesia, where recent clinical perspectives explicitly frame the issue as *balancing patient needs* with EIs while emphasising that current practice should remain focused on established best practices (Jabaudon et al., 2024).

Relatedly, while reusable materials may reduce waste, healthcare professionals have raised concerns that shifting from single-use to reusable devices or personal protective equipment could compromise patient or staff safety if decontamination, reprocessing, or infection-control practices are inadequate (Clavel et al., 2025; Vanderwee et al., 2025). Switching to low-carbon inhalers is another example and shows that healthcare’s decarbonisation has a cost that is not only financial but also ethical (Parker, 2023). It may not be clinically neutral for all patients because technique, inspiratory flow requirements, and preferences can affect adherence and symptom control, thereby creating uneven burdens across patient groups (de Maack & Dupras, 2024). More generally, sustainability-oriented change in healthcare, whether or not it increases health risks for some patients, can disproportionately affect vulnerable populations such as older adults and historically marginalised groups, thereby raising questions of equity and justice (Parker, 2025).

Taken together, these examples suggest that trade-off situations arise when emission-reduction efforts require re-evaluating what counts as acceptable benefit, risk, or safety in care. For example, in a recent cross-sectional survey of US physicians (2023–2024), 35.0% indicated that reducing healthcare’s EI can be acceptable even if it restricted treatment options of equivalent effectiveness (Hantel et al., 2025). This indicates that for a meaningful proportion of clinicians, sustainability can legitimately influence care choices among therapeutically equivalent options. Trade-off situations may become more common as broader conceptions of health (e.g. global health, One Health, Planetary Health) and decision-making processes (e.g. HTA and clinical guidelines) increasingly incorporate sustainability objectives (Bénard et al., 2025; Kuiter et al., 2025; Valera, 2025). As Holtorf et al. note, “Where trade-offs between environmental sustainability and impacts on patient benefit, social or financial factors are necessary, careful consideration and policy guidance will be required” (Holtorf et al., 2025). We argue that bioethics can play a critical role in guiding these decisions by clarifying the underlying trade-offs and making explicit the needs, values, and responsibilities that different stakeholders bring to the table.

Recent bioethics and HTA discussions increasingly treat healthcare emissions as ethically salient and call for clearer guidance on what counts as acceptable practice when environmental objectives enter clinical and procurement decisions (Bénard et al., 2025; de Maack & Dupras, 2025; Desterbecq et al., 2025; Parker, 2025). Although public health authorities and agencies, such as the Canadian Drug Agency (CDA), may eventually set quantitative benchmarks (Bénard et al., 2025), “environment-conscious HTA should also inform value-based (health, cost, and environmental outcomes) purchasing or procurement of technologies” (Holtorf et al., 2025), and define an acceptability threshold (Pegg et al., 2024). Our study contributes by examining how healthcare sustainability experts articulate these acceptability questions in concrete decision-making contexts.

To interpret experts’ accounts of potential trade-offs, we draw on work seeking to *ecologize* bioethics (Dwyer, 2009; Lee, 2017) and on theorisations of healthcare sustainability (Pierce & Jameton, 2003). A now well-recognised starting point is that mainstream bioethics has limited traction for capturing the environmental externalities of healthcare decisions, thereby tending to reproduce an anthropocentric framing of value and responsibility (Mbũgua, 2024; Prescott et al., 2022). Building on calls for a broader, “global” or environmentally attuned bioethics (Dwyer, 2009; Kadja & Rodríguez-Arias, 2024; Potter, 1988; Richie & Ehrlich, 2019), scholars have begun to reinterpret established bioethical principles to render environmental goods ethically salient (Cafaro, 2001; Munthe et al., 2021; Richie, 2019). This move is a necessary premise, but it remains insufficiently operational for the kinds of conflicts that arise when sustainability becomes an explicit objective in real-world clinical, guideline, and procurement decisions. Introducing sustainability as an explicit principle can reshape how classic bioethical principles are interpreted and prioritised, and it can also create new points of tension between these principles and the environmental considerations that global bioethics must take into account (Kovács, 2024; Resnik & Pugh, 2024). Existing approaches to sustainable healthcare ethics, or green bioethics, such as Richie and Ehrlich’s environmental bioethics principles (i.e. distributive justice, resource allocation, simplicity, and ethical economics), are valuable for advocating and guiding a sustainable transition (Richie & Ehrlich, 2019). However, they offer little operational guidance for handling conflicts *between* principles in real-world settings. Moreover, when discussed in practice, green healthcare principles remain largely confined to the clinical level, between patients and healthcare professionals (Hantel et al., 2024; Niemi et al., 2025; Richie, 2019), even though trade-offs can also arise across healthcare governance beyond care delivery (de Maack et al., 2025).

In line with hospital-level analyses that critique competing-goods framings, we treat co-benefit seeking (e.g. win–win interventions that protect environmental goods while preserving care quality) as a first-order practical orientation (Kirchhoffer & Pratt, 2025). Recognising that “*ceteris paribus*, measures with low environmental impact should be prioritised” (Valera, 2025), we rather focus on situations in which options are not equivalent (environmentally and therapeutically) and in which incorporating environmental considerations does not resolve the dilemma. When co-benefits are unavailable or contested, our analysis foregrounds how experts identify key constraints and points of blockage (e.g. up-front costs, safety requirements, quality standards, equity of access, and short- versus long-term temporalities) and propose workable negotiation strategies to justify and manage the remaining tensions.

If trade-offs are perceived as infringing on fundamental rights to appropriate care, for instance, they may generate resistance from both the public and healthcare professionals. Even though studies overall suggest substantial patient support for incorporating environmental considerations into healthcare decisions (Cohen et al., 2025; Scholz et al., 2024), heterogeneity in preferences regarding green healthcare indicates that people navigate these trade-offs in markedly different ways (Sadreghaemy et al., 2025). This suggests that some patients may not wish to consider EI when receiving care and this might be especially true when they fear that environmental priorities could come at the expense of their individual health or autonomy in making health-related decisions (Hantel et al., 2024; Kovács, 2024). This also suggests that approaches to managing trade-offs should be tailored rather than “one-size-fits-all” (Sadreghaemy et al., 2025).

Because these trade-off situations can affect what patients and professionals perceive as safe, fair, and appropriate care, their acceptability cannot be taken for granted. Consequently, environment-driven reductions in care production invite scrutiny under ethical principles (de Maack et al., 2024) and raise questions about the extent of clinician advocacy that is desirable to preserve patient trust and the legitimacy of clinician advocacy in climate-informed care (Lignou & Hart, 2024). It is to ensure buy-in for these measures, and thus their effectiveness, that these trade-offs must be analysed through the lens of stakeholders’ views and dispositions toward them. Focusing on tensions is therefore not meant to reinforce them, but rather to critically examine and, where possible, deconstruct them, as persistent false dichotomies risk legitimising the status quo and delaying the much-needed ecological transition in healthcare.

Building on this need to reflect on context-sensitive, multi-level decision support for green healthcare initiatives, this study aimed to: 1) document real and perceived ethical and social tensions that arise when sustainability objectives intersect with care priorities; 2) use high-impact climate intervention domains as a structured *elicitation device* to examine their potential impact and applicability to healthcare in the Canadian province of Québec; and 3) identify recurring, sector-specific barriers and enabling conditions that participants view as shaping the feasibility and acceptability of environmentally sustainable practices. Borrowing from Otto et al. (2020), we developed a Real-time Delphi (RTD) questionnaire based on social tipping elements (STEs) at five specific levels of intervention, identified as presenting a high chance of reducing global emissions, and conducted a 4-week online RTD consultation. This has allowed us to examine whether they were also viable at a smaller provincial scale for greener healthcare and, if not, identify sector-specific challenges. Our goal was not to test these intervention domains for general validity, but to use them to structure expert reflection and to map some of the uncertainties and coordination challenges that may condition implementation in this context. The exploration of these levels of intervention within the healthcare sector has highlighted aspects of its capacity to facilitate an environmental transition that are promising for the green healthcare research agenda. These include the sector's outdated infrastructure, its organisational culture, the scarcity of data on the EI of care, drugs, and medical devices, and the perceived incompatibility between care objectives and sustainability goals. Clarifying and, where possible, dissolving such perceived incompatibilities is a crucial step toward aligning health and sustainability imperatives.

## Method

### Real-time Delphi

Given the exploratory and prospective nature of this research, we employed the RTD consultative approach, a mixed-method research approach ideally suited to identify and anticipate issues about which there is currently little information or agreement (Belton et al., 2019). This RTD enabled us to examine specific intervention levels proposed by Otto et al. within the global context of climate change (2020). Against the backdrop of the challenges presented by climate change, and based on a wide-ranging expert consultation, their study identified social tipping interventions (STIs) (i.e. concrete levers such as removing fossil-fuel subsidies and incentivizing decentralised energy generation) designed to trigger rapid, self-reinforcing social change. The study identifies the system in which these dynamics unfold as social tipping elements (STEs) (i.e. Human Settlements (STE1)). STIs are the triggers, whereas STEs are the domains that may “tip.” In the Otto et al. study, 6 of the 12 candidate STEs were selected by the consulted experts as having the potential to address climate change, namely, information feedback, financial markets, energy production and storage, knowledge systems, norms and value systems, human settlements, and the education system. In our study, we discarded the financial markets STE, given their complexity and breadth, to prevent respondent fatigue and attrition. Each section of our questionnaire began with a brief description of the STE and its application to the healthcare setting.

- *Human Settlements (STE1):* reform laws, regulations, and building codes to promote sustainable healthcare facilities (e.g. green hospitals, carbon-neutral clinics).
- *Energy Production and Storage (STE2)*: develop low-carbon energy production and storage solutions for healthcare systems (e.g. heating, air conditioning, solar panels, batteries).
- *Education System (STE3)*: train healthcare professionals in sustainable practices (e.g. online training, seminars on sustainable healthcare, awareness of sustainable practices).
- *Norms and Value Systems (STE4)*: democratise the issue of sustainable healthcare and raise stakeholder awareness of the sector’s impact.
- *Information Feedback (STE5)*: improve stakeholder access to data on the carbon footprint and environmental impact of medicines and healthcare technologies (e.g. through product labelling).

### Panellist selection

We selected panellists with significant expertise or experience in sustainability from three expert groups: 1) health professionals involved in environmentally sustainable health issues; 2) members of government bodies or collaborative associations interested in the environmental sustainability of the healthcare sector; and 3) professionals responsible for research activities related to the ecological transition in healthcare systems. Panellists needed to practice in Québec and be able to respond to the questionnaire in French.

### Recruitment

Ethical approval was obtained on May 21, 2024, from the University of Montreal's Ethics Committee for Research in Science and Health (No. F20-CERSES-23589). Participants were then recruited over a 45-day period. CASCADES Canada (*Creating a Sustainable Canadian Health System in a Climate Crisis*), a network that supports Canada's healthcare community in transitioning to climate-resilient care, assisted with recruitment by sharing the project announcement through their weekly newsletter. We also reached out to specialised organisations, such as VITAM^1^ and contacted participants who attended the first edition of the Sustainable Health Summit (ASPQ, 2023) in Québec City. Panellists who matched one or more of the three expert categories were invited to contact the project coordinator to confirm their eligibility. Ultimately, a total of 14 individuals were selected. The sample size was deemed suitable according to the Delphi method guidelines for homogeneous groups, which recommend a panel of 10-15 participants (Duffield, 1988).

## Data collection and analysis

The 2-part questionnaire was developed by an interdisciplinary team of investigators including bioethicists, pharmacists, medical doctors, and economists. The first part was designed to characterise the socio-demographic and professional profiles of the panellists. The second part was divided into five sections, each corresponding to the STEs or *levels of intervention* from Otto et al., and aimed to:

1. Assess the potential impact and current feasibility (from very high to very low) for decarbonising the healthcare sector;
2. Anticipate possible barriers to implementing interventions related to the criterion;
3. Identify potential ethical issues and issues relating to their social acceptability; and
4. Identify possible solutions or the most promising strategies to overcome these barriers.

Panellists could explain their choice and rate their level of agreement with the other panellists’ justifications using a 5-point Likert scale (ranging from 1, strongly agree, to 5, strongly disagree). The final question explored what they considered to be promising avenues for greener healthcare research. The questionnaire was administered using the Surveylet online platform (Calibrum) (Aengenheyster et al., 2017). The 4-week data collection followed the procedure described in Masella et al. (2024).

For each question assessing potential impact and feasibility, both the status of group consensus and the strength of consensus were evaluated. The status of group consensus was categorised as either Consensus or Disagreement. Consensus was confirmed when the level of agreement reached the required threshold, as predefined in the literature, at 80.0% (Varndell et al., 2021). The strength of consensus indicates how strongly the group agrees upon the selected choice: 0% being the lowest and 100% being the maximum strength. The status of consensus was determined using the statistical mean, while the strength of consensus or group stability was calculated using the coefficient of variation. For multi-selection questions, such as question 2.1 (See the appendix), we defined consensus status (agreement or disagreement) using the majority and the percentage to calculate group stability. Thus, the consensus choice was the option that received the highest percentage of votes, and that percentage also measured the group stability. The panellists' justifications for their choice of agreement level, as well as their responses to open-ended questions, were analysed using inductive thematic analysis. After data familiarisation, we conducted initial coding to identify subthemes, which were iteratively organised into overarching themes (Braun & Clarke, 2023). For each selected verbatim excerpt, we report the panellist identification number, the number of votes supporting the statement, and the overall agreement score.

## Results and discussion

### Sample

The sample consisted of 14 participants, with an equal number of males and females. Most participants held a Ph.D. (*n* = 10). The fields in which the panellists studied were diverse, but the majority came from the biomedical and biological sciences (*n* = 11) and continued to practice in biomedical or biological science-related field (*n* = 8). Most respondents (12 out of 14) were interested in ethical and social acceptability issues related to transitioning to a more sustainable healthcare system for less than 5 years. Participant characteristics are summarised in Table 1 below.

**Table 1.** Summary of the participants' socio-demographic data

Data collected^2^	Sub-categories	Number of Panellists (n=14)
***Age***	26–35 years	4
	36–45 years	4
	46–55 years	4
	56–65 years	1
	66–75 years	1
***Sex at birth***	Male	7
	Female	7
***Belonging to a visible minority***	No	12
	Yes	0
	Prefer not to answer	2
***Highest Degree obtained***	PhD, equivalent, or higher	10
	Master’s or equivalent	4
***Years of accumulated expertise relevant to this consultation***	2 to 5 years	7
	6 to 10 years	4
	More than 10 years	3
***Years of interest in ethical and social acceptability issues related to transitioning to a more sustainable and eco-responsible healthcare sector***	≤5 years	12
	6 to 10 years	2
***Professional position or domain of expertise relevant to the consultation*** ^3^	Healthcare professionals	5
	Scientific coordinators and health-climate project managers	3
	Professors and/or researchers	6
	Management and co-management	6
	Ethics	1
***Main field of education***	Social sciences	1
	Biological and medical sciences	11
	Engineering and technology	1
	Other: Health sciences	1
***Current field of practice***	Social sciences	1
	Biological and medical sciences	8
	Public health	2
	Interdisciplinary research	2
	Other: health and social services	1

### Healthcare decarbonisation levels of intervention

Table 2 summarises the results of the quantitative questions and shows that the current potential impact of the selected levels of intervention is relatively high and, on average, higher than their feasibility levels.

**Table 2.** Summary of the main indicators for the potential impact and the current feasibility level of the five criteria of Otto et al. applied to health, and prioritisation of intervention levels to be implemented.

	Energy production and storage	Human settlements	Norms and value systems	Education systems	Information feedback
***Potential positive impact***	High	Very high	High	High	High
*Number of revisions* ^4^	18	14	16	23	15
*Group stability or level of agreement*	88,3%	88,7%	76,8%	77,5%	78,8%
*Consensus status*	Consensus	Consensus	Dissent	Dissent	Dissent
***Current level of feasibility***	Medium	Low	Medium	High	Medium
*Number of revisions*	14	15	17	18	16
*Group stability or level of agreement*	76,2%	66,7%	83,5%	86,2%	88,4%
*Consensus status*	Dissent	Dissent	Consensus	Consensus	Consensus
***Prioritisation***	47,1%	58,5%	85,7%	72,8%	35,7%

## Human settlements

### Challenges and enablers

Outdated conditions (P6, P9) of hospitals and *'the incompatibility of current infrastructure with new sustainability rules'* are challenges for the health sector (P3) pursuing human settlements. Current laws and regulations are slow to change and require strong political will, which panellists (*n* = 6) believe is lacking. As one panellist stated, *'health facilities and regulatory authorities may be reluctant to adopt major changes due to administrative complexity and the need for a thorough review of current practices'* (P4). Administrative delays complicate the implementation of new standards, particularly in the healthcare sector (P1, P7). It is recommended to address the need for change directly at the level of regulation or the building code (P1). Another type of incompatibility was highlighted regarding some infrastructure sustainability goals and clinical obligations, such as the *'need for generators to ensure continuous service'* (P6). The perception that the facility might not be in optimal condition in the absence of generators could reinforce decision-makers' reluctance to change practices regarding the use of batteries, as well as more innocuous measures such as improving thermal insulation or replacing energy-intensive equipment.

In this respect, promoting concrete examples of success can help create new norms and standardise sustainable practices in the healthcare sector (*n* = 4). The promotion of pilot projects and examples of good practice can demonstrate the feasibility and benefits of green clinics and hospitals, serving as models to encourage other facilities to follow suit (P1, P4, P11). To increase broad adoption and ensure that *'projects meet the needs and expectations of all*,*'* (P4) a diversity of stakeholders must be involved in the planning and implementation process, including healthcare professionals, facility managers, patients, and the community (P4).

The high cost of new buildings was another reported obstacle to implementing sustainable building interventions (*n* = 3). Aspects of *'financial responsibility'* (P13), particularly in private clinics *'where the building owners are not always the operators',* further complicate the situation. The importance of costs is linked to pressure from lobbies (P1, P12), making it hard to value sustainable market trajectories (Daudigeos & Valiorgue, 2010). Financial incentives, such as subsidies, are recommended (P4, P5, P8), as is mass mobilisation. By making an issue publicly visible through media coverage, social movements, or professional statements, mass mobilisation raises the reputational and electoral risks of inaction for decision-makers and can therefore sustain or legitimise regulatory action despite industry influence (Rasmussen et al., 2018; Schürmann, 2024). It also helps make the issue salient to the public and expands the coalition of affected stakeholders, thereby offsetting lobbying efforts. A massive mobilisation of all stakeholders (*n* = 5) and an *'increasing social pressure'* (P14) on decision-makers to adopt more sustainable practices could facilitate their implementation by pushing institutions to enact the necessary reforms.

## Potential impact and feasibility level

*Human settlements* were the STE with the most significant potential impact, according to the panellists, registering the highest level of stability (88,7%). Several panellists agreed with the following explanation:

"*… I believe that, in addition to being inherently beneficial for the climate, formally changing laws, standards, and regulations for the building industry would send a clear message of a paradigm shift. Not just an incentive for individuals, but a complete societal movement.*" (P7) (7 votes, level of agreement 4,1/5)

Although the overall level of feasibility was rated as low, group stability remained below 80.0%, highlighting a notable discrepancy between impact and feasibility. This is further underscored by the lowest level of consensus (66.7%) among STEs and reinforced by the challenges raised by participants, as detailed in the following section.

## Energy production and storage

### Challenges and enablers

The first challenge of energy production and storage relates to potential resistance to change from the public and healthcare professionals (*n* = 5). According to a panellist, *'healthcare establishments are often perceived as traditional and conservative environments, where structural and technological modifications are met with scepticism'* (P4). Healthcare professionals and existing systems often favour established practices, making it challenging to adopt innovations or reforms in the healthcare sector (Nilsen et al., 2020). This resistance reflects a natural reluctance to embrace change, mainly due to concerns about whether new approaches are both practical and safe, as well as whether they can be seamlessly integrated into existing clinical protocols. If professionals perceive green healthcare measures as unacceptable, they may be less likely to adopt or support them. Real and perceived trade-offs emerge from this *unacceptability*, such as *'being under-functional during peaks in energy consumption during extreme cold'* (P3), where sustainability objectives clash with the infrastructure's performance. The ease of achieving comfort and certain performance levels with fossil fuels creates an implicit opposition to decarbonisation efforts (P8, P12, P13), potentially reinforcing resistance to change. Facilitating factors, in response to potential resistance, include the integration of citizen discourse and a strong political vision, which refers to clear and consistent leadership driven by coherent, cross-government ministerial policies (P2, P5, P12). These approaches seem necessary to anticipate potential tensions, prevent conflicts, and evaluate progress.

A second challenge that was repeatedly mentioned concerned financial stakes (*n* = 9), particularly regarding the high costs of implementing new technologies, especially at remote sites. This reluctance to invest significant funds in eco-responsible projects and a *'short-term view of economic evaluation'* (P8) may impede implementation, despite some projects’ potential for long-term self-financing. Even if standards evolve faster than the projects themselves (P7), it should be possible to consider sustainability criteria, even before the planning phase of a construction project (P14). However, there may be pressure from the construction industry and investors to limit budgets and avoid additional costs, as well as ethical concerns about prioritising funding for healthcare facilities, i.e. whether resources should be allocated in priority to remote regions, disadvantaged areas, or high-use hospitals (P7).

## Potential impact and feasibility level

The consultation highlighted a growing consensus on the significant potential impact of *energy production and storage* interventions on the healthcare sector. Some initial responses were revised upwards, suggesting that participants recognise the substantial effects these interventions could have on sustainability. These revisions may reflect an understanding that, while change may seem unlikely at present, solutions at this level can nonetheless drive meaningful progress. A key insight was the necessity to *'recognise and quantify the harm caused by the way we currently do things'* first, be it in financial, environmental, social, and health terms, and over the medium and long term, as a prerequisite for effective decarbonisation (P12). Misconceptions—such as the belief that renewable technologies are prohibitively expensive or that their environmental footprint is comparable to fossil fuels — were identified as barriers (P12), emphasising the need for greater awareness to challenge these assumptions. Despite the broad recognition of their benefits, concerns were increasingly shared regarding the feasibility and the challenges of implementation:

"*I have lowered my feasibility level. I realise that there are far more obstacles than I had anticipated.*" (P7) (4 votes, level of agreement 4,2/5).

## Education system

### Challenges and enablers

On the one hand, the education system is seen as a *'weak vector for change'* (P11) and many critical opinions are expressed about the insufficiency of information and education alone (*n* = 8); on the other, some panellists are very optimistic, asserting that *'education is a method that could have positive impacts and is very feasible*, *'* and even that *'the societal impact of education on the components of sustainable health would be spectacular'* (P5). Opinions were divided, as indicated by the number of revisions (*n* = 23), which was the highest among the STEs. A conflict specific to the sector and its workers concerns priority conflicts and the demanding schedules of healthcare professionals. Indeed, while they are constrained in their choice of training courses (P6), clinical training is perceived as more essential and often prioritised over sustainable health training (P2, P6). Lack of time and workload were reported as major obstacles (P4) that are reinforced by institutional decisions favoring basic clinical training (P6). Panellists (*n* = 7) suggested making such training mandatory, with some (P7, P13) emphasising that *'professional orders'* should be responsible for implementation and codes of ethics should be revised (P7) to *'encourage healthcare professionals to become guardians of the healthcare system and the environment extending their role beyond individual patient care'* (P3).

While mandatory education programs may help raise awareness among a broad audience, they have certain limitations. For instance, they may not always focus on actions with the highest EI, or they may not effectively communicate standards of practice (P12). According to a panellist, the effectiveness of training programs is also rarely measured, making it difficult to assess their actual contribution to changes towards more sustainability (P12). In the absence of structured action plans, trained professionals can frequently revert to previous habits (P10, P12). Healthcare professionals are not typically experts in sustainability, which is an additional barrier.

Moreover, information may sometimes appear ‘*contradictory*’ (P14). For this reason, some panellists believed that education-oriented interventions should be both mandatory and provided during medical and health education (P5) to ensure uniform awareness among future professionals. This would help them internalise the principles of green healthcare before they are subjected to *'the restrictive framework of the healthcare network'* (P6). In the context of healthcare, successful implementation of sustainable interventions through educational systems also relies on the commitment of university decision-makers (*n* = 2), institutional and normative recognition that this knowledge is valuable (*n* = 2), as well as the mobilisation of students (*n* = 3).

## Potential impact and feasibility level

Most panellists, familiar with sector training practices and the level of knowledge and training of workers in their institutions regarding sustainability issues, painted a complex picture of education's strengths and limitations. Despite being considered highly impactful, opinions were particularly divided on the potential effects of interventions at the education system level. While education holds great promise as a catalyst for change (P13), it was also vividly criticised:

"*I believe that the group is generally far too optimistic about the effects of education. It is the least effective intervention of the bunch, which does not mean that it should not be done.*" (P11) (2 votes, level of agreement 3.5/5)

Many panellists reacted to the following justification, adopting a more tempered perspective, acknowledging the long-term value of education while questioning its short-term impact:

"*I indicated high feasibility but moderate impact. The comments reinforce my position. Education is relevant in the long term but has a very limited significant impact on the short and medium terms. We generally talk about continuous improvement, i.e. small incremental improvements, rather than major changes in practices.*" (P6) (6 votes, level of agreement 3.6/5)

When it came to feasibility, however, the group reached greater alignment (86,2%). Education was seen by some as relatively straightforward to implement, with training programs being among the *'easiest initiatives to organise'* (P3) and having a great potential for *'making subsequent standards easier to accept'* (P12). Others were more concerned about challenges in execution and the likelihood of immediate results. The question of how to design impactful, transformative training remains a central challenge in bridging the gap between intention and action.

## Norms and value systems

## Challenges and enablers

Concerning *norms and value systems*, the theme of resistance was predominant (*n* = 7) and took at least two forms: *social resistance*, within the general population and among healthcare professionals, and *institutional* *resistance*. For each of these levels, panellists explained the causes of this resistance, showing that the *acceptability* of greener healthcare measures varies across stakeholders and decision-making levels.

Society was described as individualistic and focused on immediate benefits, which hindered the acceptance of necessary environmental compromises (P1, P3, P12). The elderly, for example, were perceived as reluctant to limit their access to certain medical technologies, despite their EI (P3). Some panellists perceived conservative political parties as being unwilling to embrace change because of the potential adverse effects on economic growth (*n* = 2) or *'individual freedoms'* (P1). What “freedom” meant in this context was not always specified and appeared to vary across accounts, highlighting the importance of deliberation and participatory dialogue to clarify concerns and support sustainability-oriented change (P5, P8). Identifying the specific circumstances under which human rights and fundamental liberties could be affected is beyond the scope of this paper. Future research is needed to empirically map the specific circumstances under which autonomy, among other principles, could be affected.

When it comes to health professionals, they are asked to assume a dual role *'but they can only do so up to the point where it does not detract from their work'* (P7). This time, the tension stemmed from the professionals' ability to fulfil their duty, making the acceptability of such measures low. Training in the interconnectedness of health and the environment (P13) may be key to overcoming this kind of resistance and ensuring effective change. Promoting integrated sustainability frameworks, such as One Health (P13), may convince professionals that it can be done without compromising human healthcare. At the same time, there is a risk that adding ‘*pressure*’ (P9) on their shoulders may impede decarbonisation efforts (P9). Integrating sustainability objectives is also challenging for institutions that are already overwhelmed by urgent demands and limited resources (*n* = 6). Immediate priorities (P6, P10), such as *'crisis management'* and responding to an *'aging population'* (P6), often take precedence over long-term issues like sustainability.

Nevertheless, some panellists emphasised that integrating sustainability into healthcare is not just a technical or managerial issue; it also requires transforming the underlying norms and values that guide decision-making. The idea of fostering global solutions, with both local and global perspectives, was viewed as a practical and effective way to integrate sustainability within the healthcare system:

"*… this approach highlights that when the various stakeholders in the healthcare system mobilise (locally) and governments are open to change (globally), a new vision could guide the healthcare and social services system to take sustainable health into account.*" (P13).

A bottom-up approach (P8) was mentioned as a way to bridge reactive care driven by immediate needs and preventive strategies grounded in sustainability principles, allowing diverse actors to co-create shared values and ensure greater legitimacy and acceptability of sustainability measures across all levels of governance.

## Potential impact and feasibility level

The panellists’ perceptions varied widely regarding the impact of the *norms and value systems* interventions. The stability level indicates a disagreement among panellists (76,8%). Initially, potential impact was rated as high or very high but was revised downward throughout the consultation with panellists, highlighting that its immediate impact was limited (P11), despite significant efforts from advocacy groups (P7). Conversely, the feasibility of implementing specific awareness-raising actions in the short term, such as *'raising awareness among colleagues'* (P11), was considered high (83,5%). Consensus on feasibility remained relatively stable throughout the consultation, indicating agreement among panellists despite differing views on impact.

Overall, the varying perceptions among panellists suggested a complex evaluation of the effectiveness and practicality of interventions at this level. The discussion highlighted the importance of ongoing social mobilisation and the need for realistic expectations regarding the outcomes of such interventions (P6, P12):

"*I am changing my assessment from high to medium, considering the time frame. I believe that social movements can be effective, but they must be large enough to be heard by decision-makers. It is at that point that we will see significant gains. At present, we are not there yet.*" (P6) (6 votes, level of agreement 3,6/5)

## Information feedback

## Challenges and enablers

Panellists expressed significant concern about the lack of quantitative data, whether on the carbon emissions of the care life cycle, on the effectiveness of sustainable interventions already implemented, or on the avoided footprint of preventive interventions, describing it as *'incomplete or not applicable to the Québec situation'* (P6). This absence of reliable data on the ecological footprint of care was compounded by the difficulty of accurately and consistently measuring EIs (*n* = 6), which some attributed to the varying conditions under which medical products are used. Without *'standardised methods for measurement'* (P4), panellists felt decision-makers lacked the tools to make informed choices about EIs. Many expressed frustration that emissions linked to many products were still unknown (*n* = 4) and largely *'dependent on international companies, which proved difficult to regulate'* (P12). The pharmaceutical and medical sectors were frequently mentioned as being resistant for financial reasons (*n* = 5). The additional costs for the industry of *'collecting, analysing, and communicating carbon footprint information'* and *'traceability and reporting systems'* were highlighted as a significant obstacle (P4, P13), alongside concerns that revealing this information could affect sales, particularly if it painted products in an unfavourable light (P4).

It is believed that pro-environmental investments, being uncertain or involving a greater margin of risk, tend to discourage decision-making and the implementation of environmental sustainability projects (Singh & Ryvola, 2018). To some panellists in our study, making data more available would facilitate risk assessment and could help convince investors and other stakeholders that the *'actions or decisions are based on valid and socially relevant facts'* (P8). This could arguably foster corporate and social responsibility by pharmaceutical companies and medical equipment suppliers (Fagotto & Graham, 2007). It would also hold decision-makers more accountable and avoid a *'diffusion of responsibility'* (P8).

While for some panellists, the establishment of standardised norms and methodologies to measure the carbon footprint of healthcare products was essential, others (*n* = 3) suggested that different types of measures to assess the impact of interventions should also be considered (P2). Basic *'order of magnitude'* (P3) data on EIs generally appears as a valuable and practical starting point in progressing toward a *'simple, easy-to-understand rating system'* for medical equipment (P7). However, many panellists (*n* = 5) emphasised the need to avoid overly praising quantitative calculations and to foster greater interest in the development of qualitative impact scales (P12). Simplified and accessible metrics were also valued for *'the playful aspect of this information, which could be a facilitating factor for making informed choices'* (P9).

Regarding information feedback quality and impact, there was a concern that information could be *'disclosed partially or in a way that embellishes reality'* (P13). These practices, referred to as *greenwashing*, are risky, as they can undermine public trust and be counterproductive (Torelli et al., 2020; Wang et al., 2019). International organisations such as the International Organisation for Standardisation (ISO) could also *'play a key role in setting these standards'* (P4). As stated by a panellist, *'the balance between ecosystem benefits and patient risks will be at the heart of the reflection'* (P5). Yet, in this context, information could lead to *'favoring a treatment because it is less polluting, even though it might potentially result in worse disease control'* (P5). Therefore, communication directed at healthcare professionals will necessarily differ from that of patients, and the more *'personalised'* the feedback is, the easier it is for individuals to *'identify with it and successfully change their behaviours'* (P11).

## Potential impact and feasibility level

While this level of intervention seemed promising to some panellists, there was considerable disagreement in the group regarding its potential impact. Providing more and better information on the carbon cost of care may be conditionally impactful, for example, *'if combined with clear decarbonisation objectives'* (2 votes, level of agreement 4.5/5) (P12). This observation suggests that the potential impact of each STE may depend on the other STEs. In contrast, there was a strong consensus (88,4%) among panellists on medium feasibility, with the current *'lack of sufficient data to label our actions effectively'* (P3) being highlighted as an undisputed issu

## Strengths and limitations of the study

The main contribution of this study is largely qualitative and consists of an exploration of challenges and enablers to a greener healthcare sector at multiple levels. Our study provides a rich assessment of various considerations that, taken together, may give rise to ethically and socially-sensitive trade-off situations. It is, however, important to note that our study focused on decarbonisation (mainly CO_2_ reduction). While this aligns with the climate mitigation priorities of most green healthcare initiatives, a broader assessment of trade-off situations should incorporate other critical environmental issues as well, pertaining, for instance, to other greenhouse gases (e.g. CH₄, N₂O, fluorinated gases), medical waste, natural resource scarcity, biodiversity depletion, and healthcare pollution more generally (de Maack et al., 2025).

The panel comprised individuals with expertise and experience in sustainability, particularly in the healthcare sector. Most selected panellists did not have formal training in environmental sciences and sustainability beyond healthcare. Moreover, the homogeneity and relatively small size of our expert group limit the applicability of our results and conclusions to other sectors that may indirectly affect human health.

Consistent with the purpose of the Delphi method and the exploratory nature of this study, we prioritised structured expert deliberation to start identifying areas of agreement and disagreement that may arise between individuals already involved in discussions about sustainable healthcare (Hasson et al., 2025). Panellists rarely referenced Québec-specific features in their responses. Therefore, concerns and strategies that have been identified may be at least to some extent transferable to other healthcare systems facing similar sustainability challenges, conditional on appropriate contextual adaptation. The feasibility of proposed solutions will most likely depend on local conditions, especially in rural settings and among historically marginalised populations with more limited infrastructure and institutional support. Transferability should be explored in future studies that engage broader epistemic and cultural perspectives.

Panellists appeared to be influenced by the issues they face in their respective workplaces and daily practice. While this grounded the discussion in real-world concerns, it may also have introduced a form of *contextual bias*, whereby participants prioritised issues that are most salient or urgent in their immediate environment (Bénard et al., 2019), limiting the generalisability of the findings. Moreover, given that panellists were individuals already engaged with sustainability issues in healthcare, we cannot rule out the possibility of an *optimism bias* whereby the potential impact of the proposed interventions may have been overestimated (Bénard et al., 2019). This may be influenced by personal commitment or expectations, rather than evidence of feasibility

## Conclusion

The goal of this study was to gather expert perspectives on the challenges arising in the current transition to a green healthcare sector and on potential ethical trade-offs that should be addressed or resolved. Using an RTD approach, we questioned panellists on five key STEs identified by Otto et al. (2020). We found that interventions perceived as potentially the most impactful—i.e. at the levels of human settlements and energy production and storage—are also viewed as the least feasible and the most ethically and practically challenging. Perception of low feasibility might be explained by the fact that highly impactful interventions would result from action in seemingly rigid technical, economic, and regulatory contexts, necessarily requiring the involvement of various actors and stakeholders across different sectors, thereby implying significant coordination efforts (May et al., 2016).

Even when action is feasible and warranted, sector-specific characteristics of healthcare, particularly aging infrastructure and institutional rigidity, can impede change and complicate modifications to standards and practices. Perceived risks to patient safety may also affect feasibility. Similarly, the perceived trade-off between reducing EI and optimising care may undermine implementation despite growing evidence that well-designed sustainable interventions can maintain, or even enhance, care quality. Even though trade-offs more often reflect remediable institutional and procedural shortcomings rather than unavoidable moral conflicts, such perceptions could reinforce hesitation or even resistance. Conversely, interventions related to *education system*s*, norms, and value systems* were considered the most feasible. At the same time, they were the most controversial in terms of how to implement them effectively. Rather than a one-off policy, they were perceived as an ongoing, incremental process, unlike interventions focused on energy systems or infrastructure. Because their effects are harder to delimit, measure, and attribute, these interventions may be especially vulnerable to contestation and, ultimately, resistance to change. Perceived benefits can be a powerful driver of behaviour change, whereas perceived costs, whether economic or non-economic (e.g. reputational, identity-related, or professional), may hinder engagement. Making the ecological and economic benefits of sustainable choices more visible, while transparently addressing perceived costs, and reaffirming the commitment to quality care, could foster greater public support and engagement. To address *wicked problems* like climate change and sustainable healthcare, bioethics should pay attention to operational aspects and engage in the ethical reformulations we are undertaking, focusing on resistance and tensions that could hinder the transition. We see Green Healthcare Eethics as an ethic of stewardship, dedicated to advancing discussions beyond perceived trade-offs and cultivating solutions that are not only equitable but also proportionate and minimally restrictive. Documenting how conflicts are experienced, resisted, and negotiated in real-world contexts is a necessary empirical step toward developing a trade-off ethics, a theoretical effort we pursue in parallel with this current research project, and to which this paper contributes by exploring actual and prospective ethical barriers in transitioning to a greener healthcare. Future research should identify which trade-offs stakeholders deem acceptable, the conditions under which they shift, and the justificatory repertoires and decision procedures that make them legitimate across different contexts.

## Supplementary Material

Supplementary Material Appendix_questionnaire.docx

## Endnotes

^1^
A Sustainable Health Research Centre in Laval (Québec, Canada): https://vitam.ulaval.ca/

^2^
All socio-demographic characteristics are based on self-reported data.

^3^
Some participants held multiple roles, which explains why the total here does not equal n=14 participants

^4^
The number of revisions made by all survey participants to this question. A panellist may revise their opinion multiple times; each revision counts as a new one.
