# Supplementary material for: Reducing carbon emissions in the healthcare sector: a real-time delphi on challenges and enablers
Source: Glob Bioeth. 2026 May 6;37(1):2668168. doi: 10.1080/11287462.2026.2668168 (PMC13151770; doi:10.1080/11287462.2026.2668168)
Supplement: Supplementary Material — Appendix_questionnaire.docx [file RGBE_A_2668168_SM4186.docx]

**Appendix**

**Questionnaire**

**Section 1: Otto criteria applied to the healthcare sector**

| Note: Questions 1.1 to 1.5 are designed to elicit your opinion on the **potential impact** and **feasibility** of five (5) distinct levels of intervention to bring about an ecological transition in the healthcare sector. For each of these levels, you will also be asked about the **obstacles (or challenges)** to be overcome in order to implement effective interventions (i.e. favorable to the transition towards a more sustainable healthcare sector), and about possible **factors facilitating** these interventions. The sub-questions of questions 1.1 to 1.5 are therefore the same for each level of intervention. |
| --- |

- 1. **- Energy production and storage**

*Description: This level of intervention refers to the role of technological development and new modes of energy production and storage required by the healthcare system (e.g. heating, air conditioning), and the more systematic use of carbon-neutral alternatives in the energy field, where they exist (e.g. heat pumps, intelligent ventilation, heat recapture, thermal storage, solar panels, batteries).*

1. Please indicate on the scale (Likert 5 points)
   1. the **potential positive impact** you attribute to this level of intervention for a transition towards more sustainable healthcare systems

      *Very low ---------- Low ---------- Medium ---------- High ---------- Very high*
   2. the **current level of feasibility** you attribute to this level of intervention.

       *Very low ---------- Low ---------- Medium ---------- High ---------- Very high*
2. What **main obstacle or challenge** do you see for its specific application in the healthcare field? For example, do you anticipate any ethical issues or social resistance to this level of intervention?

____________________________________________________________________________________________________________________________________________________________________________________________________________________________________

1. In your opinion, what is the **main facilitating** factor associated with optimal realization of this level of intervention in the healthcare field?

____________________________________________________________________________________________________________________________________________________________________________________________________________________________________

1. Other comments : ___________________________________________________________

- 1. **- Human infrastructures**

*Description: This level of intervention refers to the possibility of modifying laws, regulations and building codes for the construction industry and public infrastructure projects, so as to eliminate the use of fossil fuels (carbon-neutral clinics) and create sustainable infrastructures (green hospitals).*

1. Please indicate on the scale (Likert 5 points)
   1. the **potential positive impact** you attribute to this level of intervention for a transition towards more sustainable healthcare systems.

      *Very low ---------- Low ---------- Medium ---------- High ---------- Very high*
   2. the **current level of feasibility** you attribute to this level of intervention.

       *Very low ---------- Low ---------- Medium ---------- High ---------- Very high*
2. What **main obstacle or challenge** do you see for its specific application in the healthcare field? For example, do you anticipate any ethical issues or social resistance to this level of intervention?

____________________________________________________________________________________________________________________________________________________________________________________________________________________________________

1. In your opinion, what is the **main facilitating** factor associated with optimal realization of this level of intervention in the healthcare field?

____________________________________________________________________________________________________________________________________________________________________________________________________________________________________

1. Other comments : ___________________________________________________________

- 1. **- Norms and value systems**

Description: This level of intervention refers to the idea of democratizing the issue of sustainable health and raising stakeholders' awareness of the impact of the healthcare sector on the environment. This can take the form (e.g.) of using government pressure or social movements, actively engaging healthcare professionals and/or their institutions, and bringing about a shift in understanding of the interconnection between human health and the environment.

1. Please indicate on the 5-point Likert scale
   1. the **potential positive impact** you attribute to this level of intervention for a transition to more sustainable healthcare systems.

      *Very low ---------- Low ---------- Medium ---------- High ---------- Very high*
   2. the **current level of feasibility** you attribute to this level of intervention.

       *Very low ---------- Low ---------- Medium ---------- High ---------- Very high*
2. What **main obstacle or challenge** do you see for its specific application in the healthcare field? For example, do you anticipate any ethical issues or social resistance to this level of intervention?

____________________________________________________________________________________________________________________________________________________________________________________________________________________________________

1. In your opinion, what is the **main facilitating** factor associated with optimal realization of this level of intervention in the healthcare field?

____________________________________________________________________________________________________________________________________________________________________________________________________________________________________

1. Other comments : ___________________________________________________________

- 1. **- Education systems**

*Description: This level of intervention refers to the education of healthcare professionals (online training, sustainable health seminars, awareness-raising on sustainable practices) in their potential commitment to actions that reduce their impact on the environment.*

1. Please indicate on the scale (Likert 5 points)
   1. the **potential positive impact** you attribute to this level of intervention for a transition towards more sustainable healthcare systems.

      *Very low ---------- Low ---------- Medium ---------- High ---------- Very high*
   2. the **current level of feasibility** you attribute to this level of intervention.

       *Very low ---------- Low ---------- Medium ---------- High ---------- Very high*
2. What **main obstacle or challenge** do you see for its specific application in the healthcare field? For example, do you anticipate any ethical issues or social resistance to this level of intervention?

____________________________________________________________________________________________________________________________________________________________________________________________________________________________________

1. In your opinion, what is the **main facilitating** factor associated with optimal realization of this level of intervention in the healthcare field?

____________________________________________________________________________________________________________________________________________________________________________________________________________________________________

1. Other comments : ___________________________________________________________

- 1. **- Information feedback**

*Description: This level of intervention refers to the significant increase in information available to actors and stakeholders on the carbon emissions and environmental impact of the medicines and other healthcare technologies they use (via, for example, the labeling of these products).*

1. Please indicate on the 5-point Likert scale
   1. the **potential positive impact** you attribute to this level of intervention for a transition towards more sustainable healthcare systems.

      *Very low ---------- Low ---------- Medium ---------- High ---------- Very high*
   2. the **current level of feasibility** you attribute to this level of intervention.

       *Very low ---------- Low ---------- Medium ---------- High ---------- Very high*
2. What **main obstacle or challenge** would you identify for its specific application to the healthcare field? For example, do you foresee any ethical issues or social resistance to this level of intervention?

____________________________________________________________________________________________________________________________________________________________________________________________________________________________________

1. In your opinion, what is the **main facilitating** factor associated with optimal realization of this level of intervention in the healthcare field?

____________________________________________________________________________________________________________________________________________________________________________________________________________________________________

1. Other comments : ___________________________________________________________

**Section 2: Prioritizing levels of intervention for a more sustainable health sector.**

2.1 Please prioritize the 6 levels of intervention discussed above:

1. Energy production and storage ;

2. Human infrastructure ;

3. Norm and value systems;

4. Education system;

5. Information feedback.

**Section 3: Another high-impact intervention for a more sustainable healthcare sector**

3.1 Based on your knowledge and expertise, can you identify another level of intervention with high impact potential to reduce the carbon footprint of the healthcare sector, or more generally, to reduce the adverse environmental consequences of the healthcare sector?

______________________________________________________________________________________________________________________________________________________________________________________________________________________________________________________

**Section 4: Promising and priority avenues for research**

4.1 Based on your knowledge and expertise, can you identify a research gap to ensure a responsible transition to a more sustainable and eco-responsible healthcare sector? For example, is there a lack of evidence on the ethical and/or social acceptability of different interventions? What types of actors or stakeholders could and should be consulted on these issues?

______________________________________________________________________________________________________________________________________________________________________________________________________________________________________________________

----------------------------------------------------------------------------------------------

***Socio-demographic questions:***

1. What age group do you belong to?
   - 18-25
   - 26-35
   - 36-45
   - 46-55
   - 56-65
   - 66-75
   - 76 or more

1. What sex were you assigned at birth?
   - Female
   - Male
   - Other :
   - I prefer not to answer

1. Do you belong to a visible minority?

- Yes
- No
- I prefer not to answer

1. What is the level of your most recent degree?

- Doctorate, equivalent or higher (3^th^cycle)
- Master's degree or equivalent (2^th^cycle)
- Bachelor's degree or equivalent (1^st^cycle)
- College
- Secondary
- Other :

1. What is your main field of training ?

- - Social sciences
  - Political science
  - Economics
  - Biological and medical sciences
  - Earth and environmental sciences
  - Public health
  - Engineering and technology
  - Mathematics, statistics and computer science
  - Interdisciplinary research
  - Other :

1. What is your current area of practice?

- - Social sciences
  - Humanities
  - Political science
  - Economic sciences
  - Biological and medical sciences
  - Earth and environmental sciences
  - Public health
  - Engineering and technology
  - Mathematics, statistics and computer science
  - Interdisciplinary research
  - Other :

1. What position (type of job, field of research, etc.) do you currently hold in relation to the theme of this consultation?

1. Please explain briefly how you feel your expertise is relevant to this consultation?

1. For approximately how long (in number of years) have you been building up the expertise you feel is relevant to this consultation?

1. For approximately how long (in years) have you been specifically interested, if at all, in the transition to a more sustainable and eco-responsible healthcare sector?

For approximately how long (in years) have you been specifically interested, if at all, in the ethical and social acceptability issues surrounding the transition to a more sustainable and eco-responsible healthcare sector?
